# Novel strip-cast Mg/Al clad sheets with excellent tensile and interfacial bonding properties

**DOI:** 10.1038/srep26333

**Published:** 2016-06-01

**Authors:** Jung-Su Kim, Dong Ho Lee, Seung-Pill Jung, Kwang Seok Lee, Ki Jong Kim, Hyoung Seop Kim, Byeong-Joo Lee, Young Won Chang, Junhan Yuh, Sunghak Lee

**Affiliations:** 1 Center for Advanced Aerospace Materials, Pohang University of Science and Technology, Pohang, 790-784, South Korea; 2 Materials Deformation Department, Light Metal Division, Korea Institute of Materials Science, Changwon, 641-010, South Korea; 3 Graduate Institute of Ferrous Technology, Pohang University of Science and Technology, Pohang 790-784, South Korea; 4 Global Technical Center, Steel Business Division, POSCO, Seoul 135-777, South Korea

## Abstract

In order to broaden industrial applications of Mg alloys, as lightest-weight metal alloys in practical uses, many efforts have been dedicated to manufacture various clad sheets which can complement inherent shortcomings of Mg alloys. Here, we present a new fabrication method of Mg/Al clad sheets by bonding thin Al alloy sheet on to Mg alloy melt during strip casting. In the as-strip-cast Mg/Al clad sheet, homogeneously distributed equi-axed dendrites existed in the Mg alloy side, and two types of thin reaction layers, *i.e*., γ (Mg_17_Al_12_) and β (Mg_2_Al_3_) phases, were formed along the Mg/Al interface. After post-treatments (homogenization, warm rolling, and annealing), the interfacial layers were deformed in a sawtooth shape by forming deformation bands in the Mg alloy and interfacial layers, which favorably led to dramatic improvement in tensile and interfacial bonding properties. This work presents new applications to multi-functional lightweight alloy sheets requiring excellent formability, surface quality, and corrosion resistance as well as tensile and interfacial bonding properties.

Mg alloys, as lightest-weight metal alloys in practical uses, have many advantages such as excellent specific strength, damping capacity, electromagnetic shielding properties, and dimensional stability^1^,^2^,^3^. However, industrial applications of Mg alloys are quite limited because of their critical shortcomings such as poor formability, surface quality, and corrosion resistance. Thus, many efforts have been dedicated to manufacture various clad sheets composed of Mg alloy and other alloys such as Al alloys or stainless steels which can effectively complement shortcomings of Mg alloys. The strip casting of Mg alloys has also been successfully commercialized by capitalizing advantages of strip casting such as rapid solidification of strip casting, reduction in micro-segregation, extension of solute solubility limit, and refining solidification structure^4^,^5^,^6^,^7^,^8^,^9^. Thus, if the fabrication method of Mg/Al clad sheets by utilizing strip casting and by cladding thin Al alloy sheet on to strip-cast Mg alloy melt can be developed, inherent shortcomings of Mg alloys can be solved.

Very few studies have been made on solidification of substrate alloy (Mg alloy) and interfacial bonding of other alloy (Al alloy) during the strip casting^10^. The interfacial bonding of strip-cast Mg/Al clad sheets should be further improved because they are not strongly bonded. Thus, interfacial reaction phases such as intermetallic compounds of γ (Mg_17_Al_12_) and β (Mg_2_Al_3_) should be optimally controlled, but effects of these parameters on interfacial bonding have not been sufficiently investigated, although trends of increased interfacial bonding properties at a certain thickness level of interfacial layers are generally accepted^11^,^12^,^13^,^14^,^15^. Here, we present a new fabrication method of Mg/Al clad sheets by strip casting followed by post-treatments (homogenization, warm rolling, and annealing). This process designing has merits of economical fabrication of Mg/Al clad sheets as well as simultaneous improvement of their interfacial bonding properties. In addition to the experimental strip casting approach, strip casting parameters are verified by a simple heat transfer calculation based on a finite element method (FEM). Interfacial bonding mechanisms are also investigated by focusing on how the kind, morphology, and thickness of interfacial reaction layers affected the Mg/Al interfacial bonding.

## Results

### FEM approach for achieving optimal strip casting parameters

The FEM is a useful computer simulation tool capable of predicting strip casting parameters. The FEM heat-transfer calculation was performed to effectively achieve optimal parameters for successful strip casting by using a commercial FEM software package, Abaqus/Explicit ver. 6.9. It was used for analyzing effects of strip casting parameters on melt pool temperatures.

**Figure 1:**
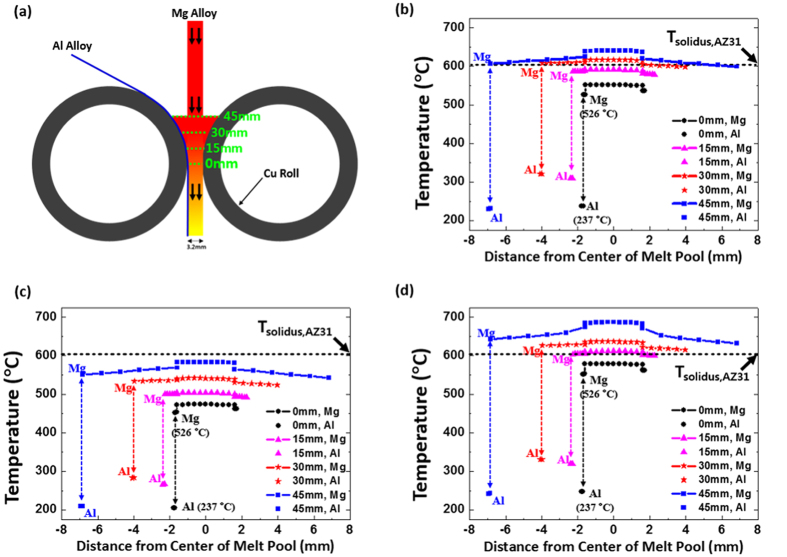
Temperature distributions of the solid Al and liquid Mg alloys. (**a**) Schematic diagram of a vertical twin-roll strip caster and (**b**) through (**d**) temperature distributions of the solid Al and liquid Mg alloys at various distances from the minimum roll gap point (the point at which the Mg alloy melt starts to escape from twin rolls) under strip casting parameters (casting temperature; 600~700 °C, roll speed; 7.8 m/min, roll gap; 3.2 mm). According to the comparison of the temperature distributions of the solid Al and liquid Mg alloys with the solidus temperature of the AZ31 Mg alloy (T_solidus,AZ31_, dashed line in **b–d**), the solidification occurs sufficiently in the border region of the Mg melt pool at optimal casting temperature of 650 °C, whereas it does not occur in the center region of the Mg melt pool at the distance of 30 mm or larger. When the temperature of the melt pool is too high, *e.g*., 700 °C, the Al alloy sheet can be melted or burned without the sufficient solidification. In the other case of the very low melt pool temperature, *e.g*., 600 °C, the Mg melt pool would be plugged by its rapid solidification between twin rolls.

Figure 1a shows a schematic diagram of a vertical twin-roll strip caster, and the melt pool region located between twin rolls is magnified. The temperature distributions of the solid Al and liquid Mg alloys are estimated at various distances from the minimum roll gap point (the point at which the Mg alloy melt starts to escape from twin rolls) under optimal strip casting parameters (casting temperature; 650 °C, roll speed; 7.8 m/min, roll gap; 3.2 mm), as shown in Fig. 1b. The solidus temperature line of the AZ31 Mg alloy (T_solidus,AZ31_) is marked by a dashed line. According to the comparison of the temperature distributions of the solid Al and liquid Mg alloys with the solidus temperature of the AZ31 Mg alloy, the solidification occurs sufficiently in the border region of the Mg melt pool, *i.e*., the region which the solid Al or liquid Mg alloy is contacted to the roll surface, whereas it does not occur in the center region of the Mg melt pool at the distance of 30 mm or larger. Figure 1c,d show the temperature distributions of the solid Al and liquid Mg alloys at casting temperatures of 600 °C and 700 °C (roll speed; 7.8 m/min, roll gap; 3.2 mm). When the temperature of the melt pool is too high, the Al alloy sheet can be melted or burned without the sufficient solidification (Fig. 1d). In the other case of the very low melt pool temperature, the Mg melt pool would be plugged by its rapid solidification between twin rolls (Fig. 1c). These temperature distribution results are well matched with the experimental results. Since the present FEM simulation is conducted under assumptions of the only heat-transfer phenomenology occurring between Mg melt pool and rolls, it cannot fully describe the detailed solidification behavior during the strip casting. Nevertheless, this FEM analysis based on simple heat transfer is a significant progress for the correct explanation of optimal strip casting parameters.

### Microstructure

**Figure 2:**
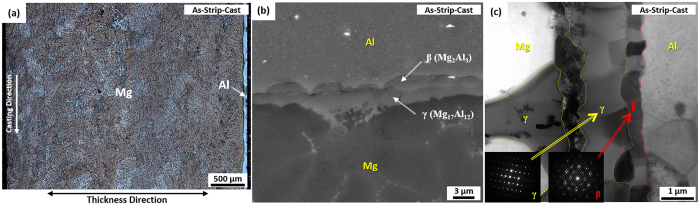
Microstructures of strip-cast Mg/Al clad sheet. (**a**) Optical micrograph, (**b**) back-scattered electron image, and (**c**) bright field TEM image of the cross-sectional area of the as-strip-cast Mg/Al sheet. The thickness of the strip-cast sheet is 3.2 mm, and the Al alloy layer is reduced from 0.1 mm to about 0.05 mm because the Al sheet is partly melted during the strip casting. Interfacial reaction layers are composed of intermetallic γ (Mg_17_Al_12_) and β (Mg_2_Al_3_) phases, which are confirmed by selected area diffraction (SAD) patterns in (**c**).

The results of our experiments are as follows. Figure 2a shows an optical micrograph of the cross-sectional area of the as-strip-cast sheet. The thickness of the as-strip-cast sheet is 3.2 mm, but that of the Al layer is reduced from 0.1 mm to about 0.05 mm because the Al sheet is partly melted during the strip casting. The Mg/Al interface is clearly observed, but does not contain any pores, cracks, or lateral delamination. The Mg alloy substrate consists of equi-axed dendrites relatively homogeneously distributed through the thickness direction, and dendrite sizes are almost similar in the top, center, and bottom areas. The columnar dendritic growth is hardly observed, which is different from microstructures of commercial AZ31 Mg alloy sheets fabricated by a horizontal twin-roll strip caster^9^,^16^,^17^,^18^.

Figure 2b shows a back-scattered electron image of the Mg/Al interface. Along the Mg/Al interface, the thin layer composed of two types of thin reaction products is found. These reaction layers are thinner than 3 μm, too thin to be observed in the optical micrograph (Fig. 2a). A bright field TEM image interfacial reaction layers are shown in Fig. 2c. Two types of reaction layers are observed along the interface as marked by dotted lines. From the selected area diffraction (SAD) patterns, reaction layers are identified as γ (Mg_17_Al_12_) and β (Mg_2_Al_3_) phases, respectively, which are characteristic intermetallic compounds given in the Mg-Al phase diagram^19^,^20^. The thickness of γ phase tends to be larger than that of β phase, which is also confirmed by Fig. 2b.

The strip-cast Mg/Al clad sheets were annealed at 300 °C for 60~120 min for the homogenization. The homogenized sheets was two-pass warm-rolled at 200 °C with total reduction ratio of 36%, and was annealed again 350 °C for 10 min in order to improve tensile properties by the recrystallization. These homogenization, two-pass warm-rolling, and additional annealing processes were used to simulate actual post-treatments (coil preheating (at 300~400 °C), reverse warm milling (at 200 °C), and short-time annealing (at 300~400 °C)) of commercial strip-cast AZ31 Mg alloys^21^,^22^,^23^. For convenience, the sheets homogenized at 300 °C for 60 min and 120 min are referred to as ‘H6’ and ‘H12’, respectively. The two-pass-rolled H6 and H12 sheets are referred to as ‘H6R’ and ‘H12R’, respectively, and the H6R and H12R sheets annealed at 350 °C for 10 min are referred to as ‘H6RA’ and ‘H12RA’, respectively.

**Figure 3:**
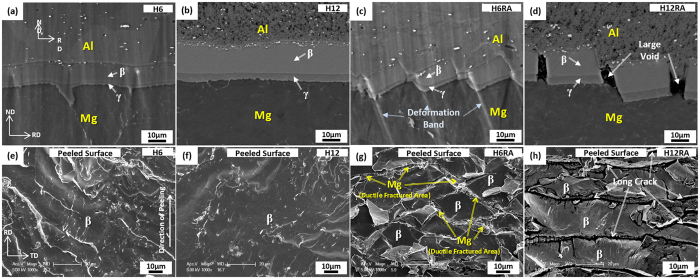
Microstructures of homogenized and two-pass warm-rolled Mg/Al clad sheets. SEM micrographs of the cross-sectional area of (**a,b**) the H6 and H12 sheets (homogenized at 300 °C for 60 min and 120 min) and (**c,d**) the H6RA and H12RA sheets (homogenized at 300 °C for 60 min and 120 min, two-pass warm-rolled at 200 °C, and annealed at 350 °C for 10 min). In the H6RA sheet, the interfacial layers are deformed in a sawtooth shape by forming deformation bands, as shown in (**b**). (**e**) Through (**h**) show SEM micrographs of the surfaces peeled along the Mg/Al interface of the H6, H12, H6RA, and H12RA sheets, respectively.

SEM micrographs of the cross-sectional area of the H6 and H12 sheets and the H6RA and H12RA sheets are shown in Fig. 3a–d. The interfacial layers, particularly the β layer, are thickened to 10.3 μm and 19.9 μm in the H6 and H12 sheets, respectively (Fig. 3a,b). In the H6RA sheet, the interfacial layers are deformed in a sawtooth shape by forming deformation bands in the Mg substrate and interfacial layers, while their thickness is retained (Fig. 3c). In the H12RA sheet, however, the interfacial layers are periodically broken to form large voids between broken layers (Fig. 3d).

**Figure 4:**
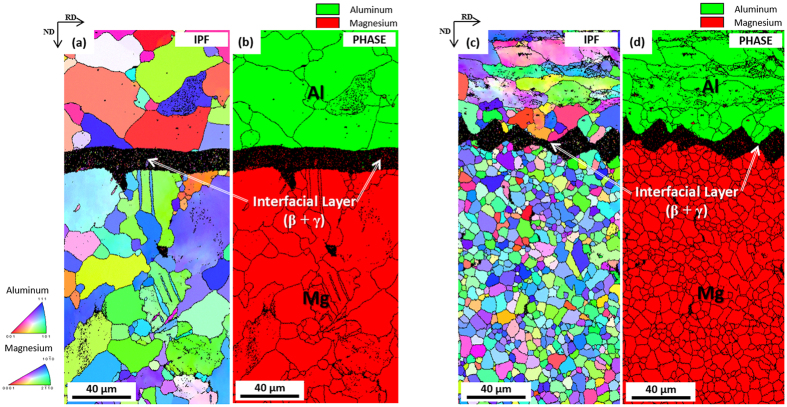
Grain refinement due to recrystallization. EBSD inverse pole figure (IPF) and phase maps, showing (**a,b**) grain coarsening in the H6 sheet and (**c,d**) grain refinement due to recrystallization in the H6RA sheet.

EBSD inverse pole figure (IPF) and phase maps of the H6 and H6RA sheets are shown in Fig. 4a–d. In the H6 sheet, most of Mg and Al grains are coarsely grown after the 300 °C-homogenization (Fig. 4a,b). In the H6RA sheet, Mg and Al grains are much smaller than those of the H6 sheet as they are refined by recrystallization after the short-time annealing at 350 °C (Fig. 4c,d), which indicates that the 350 °C-annealing is effective for the grain refinement.

### Enhanced tensile properties

Room-temperature tensile properties (yield strength, ultimate tensile strength, and elongation) are summarized in Table 1. The as-strip-cast sheet shows the high strength (264 MPa) without any plastic range. The H6 and H12 sheets have the lower strength and higher elongation than the as-strip-cast sheet. The strengths and elongation of the H6RA and H12RA sheets are greatly enhanced over the H6 and H12 sheets, respectively.

### Enhanced bonding properties

The peeling strength of the Mg/Al interface are shown in Table 1. The peeling strength of the as-strip-cast sheet is about 3.2 N/mm. The peeling strength of the H6 sheet is two and half times higher than that of the as-strip-cast sheet. When the two-pass warm rolling process is applied to the H6 sheet, the peeling strength dramatically increases up to 13.9 N/mm. The peeling strength of the H12 sheet is higher than that of the as-strip-cast sheet, but the increased peeling strength is smaller than that of the H6 sheet. Even after the two-pass warm rolling and 350 °C-annealing, the peeling strength of the H12 sheet does not increase much.

The peeled surfaces of the H6, H12, H6RA, and H12RA sheets are shown in Fig. 3e–h. The peeled surface in the H6 sheet shows the peeling in the β layer as the metallurgical bonding region formed by sufficient atomic bonding occurs (Fig. 3e), which is also confirmed by the EDS analysis. Here, the peeled surface reveals a relatively smooth fracture mode, together with some wavy patterns. The peeled surface of the H12 sheet is similar to that of the H6 sheet, but wavy patterns are hardly observed (Fig. 3f). The peeled surface of the H6RA sheet consists of flat facets originated from the β layer and ductile fracture surfaces originated from the Mg alloy substrate (Fig. 3g). In the H12RA sheet, the peeled surface is composed of flat facets originated from the β layer, which are separated by long parallel cracks as indicated by arrows in Fig. 3h. These long cracks are large voids formed from periodically broken interfacial layers (Fig. 3d). This can be confirmed by the similarity between intervals (20~30 μm) of long cracks and large voids.

## Discussion

In the present strip-cast Mg/Al clad sheet, the solidified microstructure composed of equi-axed dendrites exists in the Mg alloy substrate side (Fig. 2a). In addition, the very thin layers composed of γ and β phases are located along the Mg/Al interface (Fig. 2b,c) because the diffusion bonding time may not be sufficient during the strip casting, which results in the poor interfacial peeling strength (3.2 N/mm) (Table 1). Thus, this as-strip-cast sheet should be homogenized to recover properties of cast Mg alloy substrate and to improve interfacial peeling strength^12^,^13^,^14^,^15^.

As expected from the Mg-Al binary phase diagram^19^,^20^, interfacial layers (γ and β layers) are readily formed by the diffusion even at relatively low temperatures, and are grown with increasing homogenizing temperature or time^11^,^12^,^24^,^25^. In the H6 sheet, the interfacial layers are grown to 11.7 μm as the metallurgical bonded region expands by the interfacial diffusion, thereby leading to the improvement in interfacial peeling strength up to 7.6 N/mm. The peeled surface in the H6 sheet shows the peeling in a relatively smooth mode in the β layer (Fig. 3e). This indicates that the peeling occurs in the β layer thickly grown by the homogenizing, instead of the Mg alloy substrate, and that the peeling strength is enhanced by sufficient metallurgical bonding. When the interfacial layers are more thickly grown to 18.4 μm, like in the H12 sheet, the peeling strength is deteriorated (5.2 N/mm) as the more smoothly peeled surface appears (Fig. 3f). This is because the peeling strength is reduced as the interfacial layers are thickened over a certain thickness level, *e.g*., 10~12 μm^11^,^12^. There are various difficulties for improving the interfacial peeling strength by varying the homogenizing temperature and time only, although a considerable improvement of peeling strength is obtained from the homogenizing at 300 °C, like in the H6 sheet. In order to further improve the peeling strength by carefully controlling the kind, shape, and thickness of interfacial layers, thus, the two-pass warm rolling is essentially needed.

In the H6RA sheet, the interfacial layers are deformed in a sawtooth shape by forming deformation bands, while the interfacial layer thickness is hardly reduced by the rolling (Fig. 3c). The formation of the sawtooth-shaped interfacial layers implies that the interfacial layers composed of brittle intermetallic γ and β phases are sufficiently deformable with the nearby Mg alloy substrate by forming deformation bands without the thinning, cracking, or perforating of the interfacial layers during the warm rolling. This is because deformation bands initiated in the Mg alloy substrate are readily propagated into the interfacial layers at the warm temperature (200 °C) to deform the interfacial layers in a zig-zag mode. It was reported that an appropriate geometrical shape, instead of a simple lined or curved shape, generally enhanced the bonding properties. This occurred by reducing effects of metallurgical bond, linkage, or thermal shock arising from the interfacial bonding between hard and ductile parts in hardfacing or duo-casting of wear-resistant high-chromium cast irons^26^,^27^,^28^. Thus, the present sawtooth-shaped interfacial layers might favorably affect the interfacial peeling strength of the H6RA sheet.

In addition to the mechanical bonding by the interfacial shape modification during the warm rolling, the subsequent short-time annealing treatment at 350 °C is desirable for the additional improvement of tensile and bonding properties. The annealed microstructure is greatly refined by plastic deformation and recrystallization due to the 200 °C-warm-rolling and 350 °C-annealing, respectively. According to the tensile test results in Table 1, the strengths and elongation of the H6RA sheet are superior to those of the as-strip-cast or H6 sheet. This implies that the interfacial layers acting as embrittlement sites before the warm rolling are not brittle any more after the warm rolling followed by 350 °C-annealing. It is confirmed from the observation of the peeled surface that the peeling contains a considerable amount of ductile mode originated from the Mg alloy substrate (Fig. 3g), thereby leading to the twice higher peeling strength than that of the H6 specimen (Table 1). This enhancement in tensile and bonding properties is attributed to the modification of microstructures of the Mg/Al interface and the refined Mg and Al alloys. In the H12 sheet whose the interfacial layers are thick (19.9 μm), on the other hand, the peeling strength does not increase much even after the 200 °C-warm-rolling and 350 °C-annealing because the thick interfacial layers are easily broken during the worm rolling (Fig. 3d). The peeled surface consists of flat and smooth fracture facets separated by long parallel cracks as cracks formed between broken interfacial layers can act as nucleation sites for easy peeling along the β layer, which results in serious deterioration of peeling strength (4.6 N/mm).

**Figure 5:**
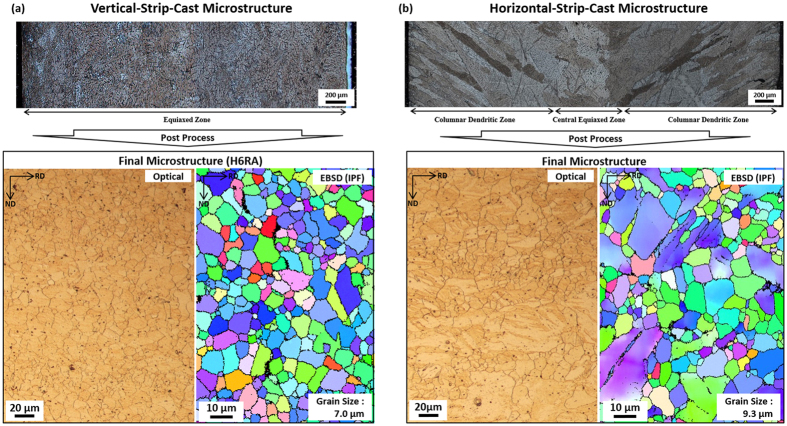
Comparison of the H6RA sheet with commercial AZ31 Mg alloy sheet. Microstructures of the (**a**) H6RA sheet and (**b**) commercial horizontal-strip-cast AZ31 Mg alloy sheet. The 2.5-mm-thick Mg alloy sheet (chemical composition; 3.0Al-0.4Mn-0.8Zn-(≤0.1)Si) was fabricated by the same processing route, *i.e*., horizontal strip casting, homogenization at 300 °C for 1 hr, reverse warm rolling at 200 °C, and annealing 350 °C for 10 min. In the horizontal-strip-cast Mg alloy sheet, dendrites are coarsely formed by the slower cooling rate during the strip casting, and the columnar growth of dendrites is observed in (**b**). Since these coarse dendrites are not sufficiently refined by the subsequent processes, the AZ31 Mg alloy sheet has larger size (size; 9.3 μm) and less homogeneous distribution of Mg grains than the vertical-strip-cast H6RA sheet (Mg grain size; 7.0 μm).

Microstructures of the H6RA sheet are compared with those of a commercial horizontal-strip-cast AZ31 Mg alloy sheet, as shown in Fig. 5a,b. The 2.5-mm-thick Mg alloy sheet (chemical composition; Mg-3.0Al-0.4Mn-0.8Zn-(≤0.1)Si) was fabricated by the same processing route, *i.e*., horizontal strip casting, homogenization at 300 °C for 1 hr, reverse warm rolling at 200 °C, and annealing 350 °C for 10 min. In the horizontal-strip-cast Mg alloy sheet, dendrites are coarsely formed, and their columnar growth is observed (Fig. 5b) because the cooling rate is relatively slow during the strip casting. Since these coarse dendrites are not sufficiently refined by the subsequent post-treatments, the AZ31 Mg alloy sheet has larger size (size; 9.3 μm) and less homogeneous distribution of Mg grains than the vertical-strip-cast H6RA sheet (Fig. 5a). In the vertical-strip-cast H6RA sheet, on the other hand, the cooling rate can be controlled to be faster than that of the vertical strip casting. Thus, and the H6RA sheet consists of fine equi-axed-shaped grains (size; 7.0 μm), and the columnar growth of dendrites is hardly observed. This modification in strip-cast microstructure of the H6RA sheet favorably affects the better tensile properties over the AZ31 Mg alloy sheet (yield strength; 185 MPa, tensile strength; 276 MPa, elongation; 13%).

**Figure 6:**
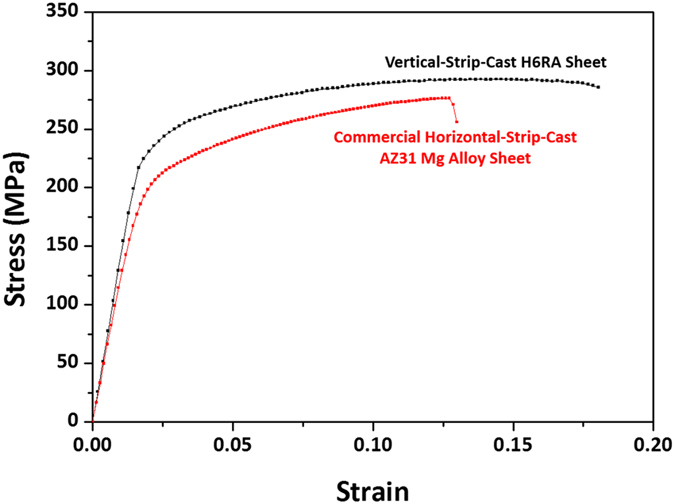
Tensile stress-strain curves of the H6RA sheet and commercial AZ31 Mg alloy sheet. Room-temperature engineering tensile stress-strain curves indicate that the H6RA sheet shows comparable or better tensile properties than the commercial horizontal-strip-cast AZ31 Mg alloy.

Considering the very thin thickness of the Al layer of the H6RA sheet (0.04 mm) in comparison to the whole thickness (2.05 mm), tensile properties of the Al layer can be negligible. The tensile test result indicates that the H6RA sheet shows comparable or better tensile properties than the commercial horizontal-strip-cast AZ31 Mg alloy (Fig. 6). This is an excellent result because the H6RA sheet contains a considerable amount of brittle interfacial layers, which are prone to the cracking, debonding, or delamination. In addition, making use of 200 °C-warm-rolling is an effective way to modify interfacial microstructures and to improve peeling strength by minimizing the deformation inhomogeneity and stress concentration.

The present strip casting and post-treatments (homogenization, warm rolling, and annealing) would prove a good way to successfully fabricate Mg/Al clad sheets having excellent tensile and interfacial bonding properties. It is also useful to understand the interfacial bonding behavior and to suggest optimal homogenizing and rolling conditions. Strip-cast Mg/Al clad sheets inevitably contain brittle interfacial layers in the Mg/Al interface, which leads to the deterioration of interfacial peeling strength, but the interfacial peeling strength is dramatically improved after the post-treatments. Since the Al layer of the clad sheets is very thin (about 0.04 mm), tensile properties of the clad sheets are mainly dependent on those of the Mg alloy substrate. Thus, the clad Mg/Al sheets can be regarded as Mg-alloy-based surface composites covered with a thin well-bonded Al layer. These results are outstanding ones, which have not been reported in previous studies on lightweight metal alloy clad sheets^11^,^12^,^24^,^29^,^30^, and the improvement of interfacial peeling strength is explained by the shape change in interfacial layers due to the formation of deformation bands with the Mg alloy substrate during the warm rolling. Since the post-treatments are actually involved in commercial fabrication processes of strip-cast Mg alloy sheets, any additional production costs as well as additional processing equipments are not needed. These strip-cast Mg/Al clad sheets have excellent tensile and interfacial properties and economic advantages as well, and thus present new applications to multi-functional lightweight alloy sheets requiring excellent formability, surface quality, and corrosion resistance as well as mechanical properties.

## Methods

### Fabrication of Mg/Al clad sheets

The AZ31 Mg alloy (composition; Mg-(2.5~3.5)Al-(0.6~1.4)Zn-(0.2~1.0Mn)-(≤0.1)Si (wt.%)) was subjected to vertical strip casting. A vertical twin-roll strip caster was composed of tundish (diameter; 72 mmφ, depth; 60 mm), crucible, twin rolls (diameter; 400 mmφ, length; 200 mm, torque; 15 hp), Al-sheet uncoiler, and brusher. The AZ31 Mg alloy was induction-melted at 625~700 °C in a crucible under an SF_6_ + CO_2_ atmosphere, and the amount of Mg alloy melt was controlled by a stopper. The alloy melt was transferred into a tundish, and then was strip-cast with a thin AISI 1050 Al alloy sheet (composition; Al-(≤0.05)Cu-(≤0.05)Mn-(≤0.05)Mg-(≤0.04)Fe-(≤0.25)Si (wt.%), thickness; 0.1 mm, width; 68 mm) to fabricate 3.2-mm-thick two-ply Mg/Al clad sheets. The Al alloy sheet was uncoiled by an uncoiler, and its surface was mechanically deoxidized by a brusher. Two side dams were installed above twin rolls to increase the height and temperature of the Mg alloy melt pool.

The roll gap was set at 3.2 mm, and the roll speed was varied at 6~24 m/min. When the melting temperature or roll speed was too slow, the Mg alloy melt was rapidly solidified before passing through twin rolls. In the case of high melting temperatures, the cast strip or Al sheet was overheated, and its surface was inhomogeneously roughened by a contraction of the Mg alloy melt or a local melting of the Al sheet. The Mg/Al interfacial bonding occurred sufficiently in the casting temperature range of 625~650 °C at the roll speed of 6~8 m/min. These casting results imply that the appropriate strip casting range is quite narrow because the sufficient time for reacting Mg melt and thin Al alloy sheet is essentially needed.

### FEM modeling

Thermal conductivities and expansion coefficients of the AZ31 Mg and 1050 Al alloys that can be used for the heat-transfer FEM analysis as well as their solidus and liquidus temperatures are summarized in Table 2. During the strip casting, heat transfer by convection in melt can occur in addition to that by conduction^31^,^32^. In this case, the amount of heat transfer may be larger than the case where the heat transfer occurs only by the conduction. In the present work, the melt convection was not considered, and only the heat conduction was considered to estimate the heat transfer as follow:


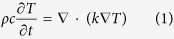


where ρ, c, k, and T are density, material specific heat, thermal conductivity, and temperature, respectively. In order to compensate for the missed convection effect, a large thermal conductivity value (500 W/m ∙ °C), instead of the value listed in Table 2, was used so that the thermal conductivity of the melt might not affect the simulation results. The thermal contact conductance is a useful parameter for the FEM simulation, from which the heat transferred from the Mg melt to Al sheet can be calculated. Since its experimental measurement is quite difficult, its optimal value is generally estimated by comparing the actual exit temperature with the exit temperatures obtained in various conditions of thermal contact conductance^33^. The thermal contact conductance estimated in the present study is 3000 W/m^2^ ∙ °C, and is used in the FEM simulation.

### Microstructural characterization

These Mg/Al clad sheets were sectioned and mechanically polished with a diamond paste (size; 1 μm) before observing microstructures of longitudinal-short-transverse (L-S) plane by an optical microscope and a scanning electron microscope (SEM, model; JSM-6330F, JEOL, Japan). Phases in the Mg/Al interface were analyzed by an energy dispersive spectroscopy (EDS). Electron back-scatter diffraction (EBSD) analysis was also conducted by a field emission scanning electron microscope (FE-SEM, model; Helios Nanolab^TM^, FEI, USA) to obtain inverse pole figure (IPF) and phase maps. Specimens for transmission electron microscopy (TEM) observation were prepared by a focused ion beam (FIB, model: Quanta 3D FEG, FEI, USA) technique. Thin FIB specimens were observed by a TEM (model; JEM-2100F, JEOL, Japan) at an acceleration voltage of 200 kV.

### Mechanical property tests

Plate-type tensile specimens (gage length; 20.0 mm, gage width; 5.0 mm, gage thickness; 3.2~2.05 mm) were prepared in the longitudinal direction. They were tested at room temperature at a strain rate of 10^−3^ s^−1^ by a universal testing machine (model; Instron 1361, Instron Corp., Canton, MA, USA) of 100 kN capacity, in accordance with ASTM E 8/E 8M standard specification^34^.

For roller-drum peel tests, plate-type specimens (100 × 10 × 2 mm) were prepared in the longitudinal direction. They were tested at room temperature at a cross-head speed of 6 mm/min by using a screw-driven universal testing machine (model; 8861, Instron, Canton, MA, USA) of 100 kN capacity. The peeling strength of Mg/Al interface was measured in accordance with ASTM-D3167 standard specification^35^. After the peel test, the peeled surface was observed by an SEM.

## Additional Information

**How to cite this article**: Kim, J.-S. *et al.* Novel strip-cast Mg/Al clad sheets with excellent tensile and interfacial bonding properties. *Sci. Rep.* **6**, 26333; doi: 10.1038/srep26333 (2016).

## Figures and Tables

**Table 1:** Thickness of interfacial layers, tensile properties, and peeling strength of the strip-cast Mg/Al clad sheets.

Clad sheet	Thickness of γ layer (μm)	Thickness of β layer (μm)	Yield strength (MPa)	Tensile strength (MPa)	Total elongation (%)	Peeling strength (N/mm)
As-strip-cast	1.30 ± 0.19	1.03 ± 0.91	243.7 ± 8.5	263.6 ± 11.4	2.2 ± 0.32	3.2 ± 0.6
H6	1.95 ± 0.02	9.16 ± 0.26	146.9 ± 4.3	204.8 ± 8.2	7.6 ± 0.88	7.6 ± 1.1
H12	4.11 ± 0.18	15.77 ± 1.25	149.7 ± 3.6	189.2 ± 9.1	7.9 ± 0.93	5.2 ± 1.4
H6R	1.62 ± 0.34	7.63 ± 1.94	264.6 ± 5.5	307.5 ± 6.3	4.2 ± 0.78	13.9 ± 1.4
H12R	3.52 ± 0.08	14.71 ± 1.55	226.0 ± 4.9	292.0 ± 7.2	7.6 ± 0.28	4.7 ± 1.0
H6RA	1.70 ± 0.10	7.69 ± 2.25	219.4 ± 2.1	292.7 ± 5.5	18.0 ± 1.02	13.7 ± 0.7
H12RA	3.97 ± 0.27	15.01 ± 1.97	188.0 ± 6.7	291.3 ± 3.7	17.8 ± 1.32	4.6 ± 1.1

**Table 2:** Solidus and liquidus temperatures, thermal conductivity, and thermal expansion coefficient of the AZ31 Mg and 1050 Al alloys^25^,^27^.

Alloy	Solidus temperature (°C)	Liquidus temperature (°C)	Thermal conductivity (W/m ∙ °C)	Thermal expansion coefficient (°C^−1^)
AZ31	605	630	96	2.64 × 10^−5^
Al1050	646	657	222	2.40 × 10^−5^
